# Shifting norms, static behaviour: effects of dynamic norms on meat consumption

**DOI:** 10.1098/rsos.240407

**Published:** 2024-06-26

**Authors:** Alaa Aldoh, Paul Sparks, Peter R. Harris

**Affiliations:** ^1^ Department of Psychology, University of Amsterdam, Amsterdam, The Netherlands; ^2^ School of Psychology, University of Sussex, Bighton, UK

**Keywords:** dynamic norms, sustainability, norms, communication, visual, meat

## Abstract

While decreasing their meat consumption is one of the most impactful behaviours an individual may carry out to reduce their carbon emissions, it is still a minority behaviour in many parts of the world. Research suggests that communicating information about changing ‘dynamic’ norms may be a useful tool for changing attitudes and behaviours in the direction of those currently held by the minority. This study uses a 2 × 2 mixed design (norm type [dynamic/static] × visual cue [present/absent, and a no-task control), and a follow-up assessment after one week to investigate the effect of making dynamic norms salient on various meat consumption outcomes: attitudes towards meat consumption, interest in reducing one’s own meat consumption, intentions to reduce one’s own meat consumption and self-reported meat consumption. We used an online sample of British participants (*N* = 1294), ranging in age 18–77 (*M*_age_ = 39.97, s.d._age_ = 13.71; 55.8% female). We hypothesized that: (i) dynamic norms will positively influence meat consumption outcomes; (ii) visual cues will accentuate the difference between norm conditions; (iii) using a visual cue will enhance the effect of dynamic norms; and (iv) any effects of dynamic norms will endure over a period of one week. We found no positive effect of dynamic norms (versus static norms) on any outcome at time 1, and no positive effect on changes in outcomes from time 1 to time 2. However, we found a positive interaction of norm type and visual cue at time 1 (although not from time 1 to time 2): the addition of a visual cue to dynamic norm messages enhanced the positive effect of the message at time 1 (but did not enhance the changes occurring from time 1 to time 2). Analyses for changes in self-reported meat consumption did not reach our evidential threshold. We discuss the practical and theoretical implications of these findings.

## 1. Introduction

Meat production is a major contributor to greenhouse gas emissions and environmental degradation, and decreasing consumption of meat is considered a high-impact action for reducing carbon emissions [1]. A review of educational textbooks and government resources for civilians from the EU, USA, Canada and Australia found that there tends to be a focus on behaviours with low impact on the environment, rather than on effective emission reduction strategies that can narrow the climate mitigation gap [2]. Even if individuals are knowledgeable of highly impactful emission-reducing behaviours, and are willing to align their behaviour with climate targets, social norms may act as a barrier to the uptake of environmentally sustainable behaviours if the current norms reinforce unsustainable behaviours [3,4].

### 1.1. Social norms for behaviour change

Many food choices are shaped by habits triggered by situational cues that lie largely outside people’s conscious awareness [5,6]. People rely on the social context to infer what is acceptable behaviour, and dietary behaviour is often related to perceptions of normative behaviour in peer groups [7]. Social norms may be a promising target for changing habitual eating behaviour especially perhaps as they may sometimes bypass conscious motivations in their influence on behaviour [8–10]. In line with the focus theory of normative conduct [11], portraying desired behaviour (e.g. decreasing meat consumption) as aligned with injunctive or descriptive norms can shift existing behaviours [7,12].

Descriptive norms refer to the perceived prevalence of a behaviour, whereas injunctive norms refer to perceptions of expected behaviour [11]. In a series of experiments, Jacobson *et al.* [12] demonstrated that descriptive and injunctive norms differ in the motivational forces that influence conformity to each type of norm. Injunctive norms are said to relate both to the intrapersonal goal of accuracy/efficiency, and to the interpersonal goal of social approval. This is thought to evoke thoughts about dual (and potentially duelling) goals, leading to the experience of decision-making conflict, and greater capacity for effortful self-regulation to follow. On the other hand, descriptive norms are said to provide a cue for accurate or efficient behaviour; they do not necessarily invoke a sense of social obligation (in contrast to injunctive norms). This in turn is associated with an accuracy/efficiency goal of choosing correctly only, leading to lesser degree of decision-making conflict, thereby requiring less effortful self-regulation to resist rather than follow. Descriptive and injunctive norms differentially affect behaviour to the extent that each norm is currently salient, even when the norms advocate the same behaviour [12]. Research also suggests that injunctive norms influence behaviour differently in varied cultural contexts, whereas descriptive norms do not. For example, differing injunctive norms—but not descriptive norms—contributed to cultural variations in individuals’ level of discomfort caused by incivility in high- and low-ranking perpetrators in a study comparing Korean and British cultures that differ in power distance (i.e. the degree to which a culture accepts or legitimizes power imbalances and hierarchical disparities [13]. Manipulating either type of norm typically influences behaviour, and a recent review suggests that injunctive norm manipulations have a stronger meta-analytic effect on behaviour than descriptive norms [14]. However, it is worth noting that the design of that meta-analysis did not involve a controlled comparison of the two types of norms. A recent meta-analysis of 100 articles comparing the influence of injunctive norms, personal norms and descriptive norms on conservation behaviour found that personal and descriptive norms had a larger relative influence on behavioural intentions and were more often significantly associated with intentions than were injunctive norms ([15]; see also [16]). In instances in which the desired behaviour is not aligned with norms, or is even contrary to existing norms, portraying a descriptive increase in the minority behaviour can increase people’s conformity to what they perceive to be a future descriptive norm [17,18]. For example, Cheng *et al.* [19] found that female students in high school and college who were exposed to information about an increasing number of women pursuing STEM careers reported higher interest in STEM careers and intentions to enter STEM fields themselves.

Although meat eating is still widespread, in many parts of the world people’s dietary behaviours are changing. For example, in the UK, meat consumption is declining and the proportions of vegans and vegetarians have both doubled in the 20 years prior to 2002 [20]. In 2016, a third of British survey respondents reported eating less meat than a year before data collection [21]. Average meat consumption per capita per day in the UK decreased between 2008 and 2019 [22]. Similar trends have been observed in Canada, where consumption of beef, pork and veal per capita has reduced from 1980 to 2020 (34.1%, 41.42% and 35.25% reductions, respectively [23]). Similarly, a 2016 survey reports one-third of Americans were eating less meat than they were 3 years previously [24]. Interestingly, 2019 Gallup survey found that while 23% of Americans reported eating less meat in the previous year, 5% had reported eating more meat [25]. Although there appears to be a growing minority of people limiting their meat consumption, only a much smaller minority are actually increasing their meat consumption.

Psychological studies using descriptive social norms have typically used ‘static’ norm framings communicating current levels of normative attitude/behaviour. Whereas people typically act in line with static descriptive norms when they are performed by the majority, salient static minority norms may have the opposite effect on desired behaviour change [26]. That is, static minority norms may reduce engagement in the desired behaviour. Therefore, in the past decade, researchers have been ‘leveraging’ changing norms to instigate attitude and behaviour change to reduce meat consumption [8,10]. By making dynamic norms (i.e. descriptive norms that are changing) salient, people can begin to conform to behaviour that is on the rise, even if it is not currently the prevailing norm. In contrast to ‘static’ descriptive norm messages, which communicate current norms, dynamic descriptive norm messages draw attention to change or trend in the norm over time [4]. For example, a static descriptive norm would refer to information about current normative behaviours and beliefs of others (e.g. ‘most people eat meat’ or ‘some people currently limit eating meat’). Alternatively, a dynamic descriptive norm would refer to changes in normative behaviours or beliefs over time (e.g. ‘more and more people are limiting eating meat’).

Several studies have demonstrated the effect of dynamic norms on interest in reducing meat consumption. In a series of online studies, Sparkman and Walton [17] demonstrated the efficacy of using dynamic norm information to increase interest in reducing meat consumption. They extended their findings to a field study conducted in a university campus café where dynamic norm information doubled the percentage of patrons who ordered a meatless lunch compared with static norms. In a series of cross-sectional and quasi-experimental studies examining the influence of personal norms on the effectiveness of social norm interventions, de Groot *et al.* [27] found that both dynamic and static norm information were more effective than the weaker one’s personal norms towards the pro-environmental behaviour. It is unclear whether there was a difference between dynamic and static norms as differences were not tested (see Study 1) or directly compared in the study design (see Studies 2 and 3). They also found that dynamic norm information depicting 80% of the Dutch population reducing their meat consumption influenced people more strongly than did minority dynamic norms depicting a change in only 20% of the Dutch population. Stea and Pickering [28] tested six messages presenting information about the environmental impacts of meat production varying in social norm representation (social norm present or absent) and identity salience (Canadian place identity, global place identity or none). They found that including social norm aspects depicting changing diets (e.g. ‘people are making dietary changes to reflect their feelings towards these impacts’) in the message did not result in significant differences in intentions to reduce meat consumption compared with the control condition. Aldoh *et al.* [29] conducted a study investigating the effect of dynamic norms on cognitive factors related to meat consumption, and found no difference between the dynamic norm condition and a static norm control condition in interest, attitudes and intentions to reduce meat consumption. Another recent study found no comparative advantage of using dynamic norms compared with static norms or no normative messages in influencing intentions to eat less red meat [30]. Interestingly, those latter authors did find a significant interaction between dynamic/static norm messages and perceptions of future approval of behaviour, where intentions to eat less red meat were higher when participants expected others to approve of this behaviour in the future. In a series of four field experiments, Sparkman *et al.* [31] found mixed effects of dynamic norm messages on meatless orders. Although dynamic norm messages ‘modestly’ increased orders of vegetarian dishes, some studies were underpowered and effects were not always significant. In one study, dynamic norm messages appeared to backfire, leading to a decrease in order of vegetarian dishes at a fine-dining Italian restaurant ([31], Study 4). Altogether, the reported results point towards a positive effect of dynamic norm interventions when outcomes are measured immediately after intervention, but this positive effect is not always present.

Dynamic norms have also shown promising effects over the long term. For example, Macdonald *et al.* [32] tested two types of dynamic norm appeals compared with a control group: (i) an appeal to reduce meat consumption or (ii) an appeal to eliminate meat consumption entirely. They found that both dynamic norm appeals were effective in reducing reported meat consumption five weeks from intervention, but there were no significant differences between the ‘reduce’ and the ‘eliminate’ appeals. A later study conducted by Sparkman *et al.* ([33], Study 1) used a similar design comparing ‘reduce’ and ‘eliminate’ appeals each against a control condition. Interestingly, they found that only the ‘reduce’ dynamic norm appeal successfully decreased participants’ self-reported consumption relative to the control condition for the five months duration of the study. However, in a follow-up study using a representative sample of the US population, neither the ‘reduce’ nor ‘eliminate’ appeals were successful in changing reported meat consumption over time ([33], Study 2). Their results suggest that the dynamic norm appeal to reduce meat consumption is effective in a subsample matching their initial study’s sample demographics, which was generally younger (less than 60 years old), more liberal (very liberal to conservative) and more educated (have at least some college education). This echoes findings of past research showing that young adults and old adults may be more susceptible to attitude and behaviour change [14,34]. Amiot *et al.* [35] designed a multi-component intervention (to reduce meat consumption) consisting of five elements: (i) social norm component; (ii) informational/educational component; (iii) appeal to fear; (iv) mind-attribution induction; and (v) goal setting/self-monitoring component. The social norm component included a presentation describing changing social norms regarding reduced meat eating since 1980 in Canada. The intervention resulted in significant decreases in total red meat consumption four weeks from baseline. Although the intervention was effective, it is difficult to ascertain which component (or combination of components) drove the effects.

Research using information about changing norms varies considerably in its implementation, and the diverging results of past studies leave many unanswered questions about the factors affecting the strength of dynamic norm messaging. Although research using social norm manipulations is generally effective in influencing perceived norms, attitudes, intentions and behaviour, there is a large degree of heterogeneity among effect sizes, suggesting that other study characteristics lead to variations in findings [14]. Further research on the modes of communicating normative information should help improve understanding of the optimal ways of communicating dynamic norms.

### 1.2. Communicating a change in norms

Although dynamic norm research is quickly growing, there is still uncertainty about the more effective ways of making dynamic norms salient in order to adequately assess their influence on behaviour and its antecedents. With growing awareness of changing meat-eating trends in the UK, it is also unclear if experimental manipulations making dynamic norms salient fail due to the ineffectiveness of dynamic norms in a given context, or because a dynamic norm is salient even in control conditions without experimental manipulation. Manipulating dynamic norms experimentally involves making the change in norms salient. However, it is possible that study participants are already aware of dynamic norms in the absence of any experimental manipulation. For example, Aldoh *et al.* [29] found that the majority of participants in a pilot study were already aware of changing meat-eating norms in the UK, and upwards of 80% of participants in the main study were expecting a future decrease in meat consumption.

To test the effectiveness of dynamic norm information, it is probably beneficial to use control groups in which a static (unchanging) norm is similarly made salient. This makes it possible to discern that the dynamic aspect of the information influences the measured outcomes, as opposed to any normative information. Rhodes *et al*.’s [14] review found that a control condition making a weaker or opposing norm salient was the most effective for demonstrating the effects of tested norm manipulations.

When considering the method of delivering normative information, studies have previously used a number of modes such as text narrative, video, images and face-to-face individual or group intervention. Although the most prevalent method has been through written text, studies examining the effect of descriptive norms on behavioural outcomes using multiple modes of delivery outperformed any other mode by itself. One possible way to increase the salience of the dynamic norm experimentally is to use visual cues to depict the norm. Sparkman and Walton’s ([17], Study 3) research uses this in comparisons between three groups where text prompts were supplemented with line graphs depicting a dynamic norm, and a pie chart depicting a static norm. Those authors found a difference between a dynamic norm condition depicting future growth in people’s decreasing meat consumption using a line chart, and a static norm condition depicting the current prevalence of people’s decreasing meat consumption using a pie chart. Whereas a pie chart is useful in showing the current distribution of the norm, it is less useful in portraying the unchanging nature of the static norm. A visual cue depicting a stable trend in the static norm, or increasing trend in the dynamic norm, can increase the distinctiveness of dynamic norms. Visual cues may also be potentially useful in increasing engagement with the information provided, thereby increasing the effectiveness of the manipulation used. In a similar vein, Vasiljevic *et al.* [36] cite the use of pictorial presentations of injunctive norms as a possible reason for diverging results of injunctive norms on healthy food choice compared with an earlier study using textual presentation of injunctive norms, which found no effect of injunctive norms on food choice (cf. [37]).

### 1.3. The current study

The present study investigates the effectiveness of dynamic norm information in the context of reducing meat consumption. Specifically, we are interested in testing the following hypotheses:

Hypothesis 1: Making information about dynamic norms (in relation to reduced meat consumption in the UK) salient will lead to more positive effects on meat consumption outcomes than does making static norm information salient.

H1a. Participants who view dynamic norm information will have more positive *attitudes* at T1 toward reducing their meat consumption compared with participants who view static norm information.

H1b. Participants who view dynamic norm information will report higher *interest* at T1 in reducing their meat consumption compared with participants who view static norm information.

H1c. Participants who view dynamic norm information will report higher *intentions* at T1 to reduce their meat consumption compared with participants who view static norm information.

Hypothesis 2: Including a visual cue will accentuate the difference between the dynamic norm and static norm conditions in meat consumption outcomes.

H2a. Visual cues will accentuate the difference between dynamic and static norm conditions in participants’ *attitudes* toward reducing meat consumption.

H2b. Visual cues will accentuate the difference between dynamic and static norm conditions in participants’ *interest* in reducing their meat consumption.

H2c. Visual cues will accentuate the difference between dynamic and static norm conditions in participants’ *intentions* to reduce their meat consumption.

Hypothesis 3: Including a visual cue will lead to a greater effect of dynamic norm information on meat consumption outcomes.

H3a. Participants who view dynamic norm information accompanied by a visual cue will have more positive *attitudes* toward reducing their meat consumption compared with participants who view dynamic norm information without the visual cue.

H3b. Participants who view dynamic norm information accompanied by a visual cue will report higher *interest* in reducing their meat consumption compared with participants who view dynamic norm information without the visual cue.

H3c. Participants who view dynamic norm information accompanied by a visual cue will report higher *intentions* to reduce their meat consumption compared with participants who view dynamic norm information without the visual cue.

Hypothesis 4: Dynamic norm information will positively influence meat consumption outcomes over a period of one week.

H4a. There will be a greater positive change in participants’ *attitudes* toward reducing meat consumption in the dynamic norm conditions compared with the static norm conditions over a period of one week.

H4b. There will be a greater positive change in participants’ *interest* in reducing their meat consumption in the dynamic norm conditions compared with the static norm conditions over a period of one week.

H4c. There will be a greater positive change in participants’ *intentions* to reduce their meat consumption in the dynamic norm conditions compared with the static norm conditions over a period of one week.

H4d. Participants in the dynamic norm conditions will reduce their self-reported *meat consumption* more than participants in the static norm conditions over a period of one week.

Hypothesis 5: Including a visual cue will accentuate the difference between the dynamic norm and static norm conditions in meat consumption outcomes over a period of one week.

H5a. Visual cues will accentuate the difference between dynamic and static norm conditions in participants’ *attitudes change* regarding reducing meat consumption (between time 1 to time 2).

H5b. Visual cues will accentuate the difference between dynamic and static norm conditions in participants’ *interest change* in reducing their meat consumption (between time 1 to time 2).

H5c. Visual cues will accentuate the difference between dynamic and static norm conditions in participants’ *intentions change* to reduce their meat consumption (between time 1 to time 2).

H5d. Visual cues will accentuate the difference between dynamic and static norm conditions in participants’ self-reported *meat consumption* (between time 1 to time 2).

Hypothesis 6: Including a visual cue will increase the effect of dynamic norm information on meat consumption outcomes over a period of one week.

H6a. Participants who view dynamic norm information accompanied by a visual cue will show more positive *attitude change* regarding reducing their meat consumption compared with participants who view dynamic norm information without the visual cue (between time 1 to time 2).

H6b. Participants who view dynamic norm information accompanied by a visual cue will show more *interest change* in reducing their meat consumption compared with participants who view dynamic norm information without the visual cue (between time 1 to time 2).

H6c. Participants who view dynamic norm information accompanied by a visual cue will show higher *intentions change* to reduce their meat consumption compared with participants who view dynamic norm information without the visual cue (between time 1 to time 2).

H6d. Participants who view dynamic norm information accompanied by a visual cue will reduce their self-reported *meat consumption* more than participants who view dynamic norm information without the visual cue (between time 1 to time 2).

## 2. Pilot study

We present here the results of an initial study investigating the effect of dynamic norms on meat consumption using visual cues. The study was conducted to test the materials used and to estimate the influence of dynamic norms using a visual cue (versus static norms with a visual cue) on measured outcomes. The study included two conditions: a dynamic norm prompt with a visual cue and a static norm prompt with a visual cue. For the pilot study, we hypothesized that dynamic norms will positively influence meat consumption outcomes relative to static norms. All relevant study materials, data, and analyses are publicly hosted on Open Science Framework (OSF; https://osf.io/qe739/).

### 2.1. Participants

A total of 1075 participants took part in the study online through Prolific (https://www.prolific.co; a participant recruitment website). Seventeen participants were excluded from the sample as they were vegan or vegetarian, and 16 were excluded for starting, but not completing the survey, resulting in 1042 participants. Using a robust Mahalanobis distance based on the minimum covariance determinant [38,39], 144 multivariate outliers were detected and removed. The final sample included in the analyses (*n* = 898) ranged in age from 18 to 80 years (*M*_age_ = 36.44, s.d. = 13.45). The participants were predominantly female (55.68%). They received £0.25 for successfully completing the 2–3 min task. The rate of payment was £5.60/hr. We intended to collect data until a threshold of *B* > 5 or *B* < 1/5 was reached for the primary hypotheses. After collecting data from over 1000 participants, however, we had still not reached the threshold for all measured outcomes, but we terminated data collection due to funding limitations. A randomization check revealed no systematic differences between conditions in age, gender, political position and home country (all *p*s > .05).

### 2.2. Procedure and materials

Participants were recruited from Prolific and were redirected to a survey hosted on Qualtrics (https://www.qualtrics.com). We used Prolific’s pre-screening criteria to exclude participants who were not eligible. Specifically, we did not make the study visible to participants who (i) were not UK nationals; (ii) followed a vegan/vegetarian diet; and (iii) had previously participated in other related studies conducted by the primary author. Participants were randomly allocated to one of two conditions: (i) dynamic norm with visual cue or (ii) static norm with visual cue. Then participants completed single-item measures of interest in reducing meat consumption (‘I am interested in eating less meat’, 0 = *not at all interested* to 100 = *extremely interested*), attitudes towards reducing meat consumption (‘My attitude towards eating less meat is…’, 0 = *extremely unfavourable* to 100 = *extremely favourable*), expectations to reduce own meat consumption (‘I expect to eat less meat within the next year’, 0 = *strongly disagree* to 100 = *strongly agree*) and intentions to reduce meat consumption (‘I intend to eat less meat within the next year’, 0 = *strongly disagree* to 100 = *strongly agree*). Measures of expectations and intentions were later combined into a composite measure of expectations and intentions due to the large intercorrelation between them (*r* = .97). Participants also provided estimates of people who are currently/will be reducing their meat consumption now, next year and 6 years from now. Finally, participants answered some demographic questions, and the study was concluded.

### 2.3. Results

Means, standard deviations and intercorrelations between measured study variables are reported in table 1. We used a path analysis to test differences between conditions in measured meat consumption outcomes (see table 2 for results). Participants in the visual dynamic norm condition reported higher scores on the composite measure of intentions and expectations to reduce their own meat consumption than did participants in the visual static norm condition (visual dynamic: *M* = 51.63, s.d. = 32.84; visual static: *M* = 46.41, s.d. = 31.94; *B*_HN(0,5%)_ = 7.06, RR [2.2, 10.2]). However, there was no evidence for or against the presence of a difference between conditions in interest in reducing meat consumption (visual dynamic: *M* = 55.11, s.d. = 34.24; visual static: *M* = 50.97, s.d. = 34.27), or attitudes towards reducing meat consumption (visual dynamic: *M* = 60.55, s.d. = 27.21; visual static: *M* = 56.73, s.d. = 26.87).

**Table 1.** Descriptive statistics and intercorrelations (pilot study).

measure	*M*	s.d.	correlations					
			1	2	3	4	5	6
interest	52.99	34.30	—					
attitude	58.59	27.09	.65**	—				
intention	49.42	32.83	.92**	.65**	—			
expectation	48.50	32.55	.91**	.64**	.97**	—		
avg. intention + expectation	48.96	32.47	.92**	.64**	.99**	.99**	—	
own consumption	4.65	1.85	−.41**	−.34**	−.46**	−.47**	−.47**	—
projected consumption	42.13	14.13	.33**	.19**	.34**	.35**	.35**	−.14**

Note: *N* = 898.

** *p* < .01.

**Table 2.** Differences between conditions in meat consumption outcomes (pilot study).

*item*	*b* (%)	s.e.	*p*	95% CI	inference		
					*B*_HN(0,5%)_	RR	conclusion^a^
interest	4.15	2.28	.069	[−0.33, 8.62]	3.08	[0.05, 15]	None
attitude	3.82	1.80	.034	[0.29, 7.36]	4.83	[4.6, 15]	None
intention	5.48	2.18	.012	[1.20, 9.75]	11.03	[1.8, 15]	H_1_
expectations	4.96	2.17	.022	[0.71, 9.20]	7.06	[2.2, 10.2]	H_1_
average intentions + expectations	5.22	2.16	.016	[0.98, 9.45]	9.09	[1.9, 14.6]	H_1_

Note: *N* = 898.

^a^ H_0_ = evidence for null hypothesis, None = no conclusion, H_1_ = evidence for alternative hypothesis

*b*, raw regression slope; CI, confidence interval; RR, robustness region.

### 2.4. Conclusion

The results suggest a positive effect of dynamic norms on intentions/expectations to reduce own meat consumption. Only the effect of dynamic norms on intentions and expectations to reduce meat consumption provided sufficient evidence for the alternative hypothesis. Furthermore, it is not clear if using visual cues for each condition drove the effects found, or if the messages would be equally effective using text alone. It is also unclear if the effects persist over a longer period of time and if there would be effects on actual reported behaviour. Research suggests that the effects of norm manipulation on behaviour tend to dissipate over time, but time passed since message exposure did not influence attitudes, intentions or perceived injunctive norms [14]. Using one-time snapshots of outcomes makes it difficult to make claims about the long-term influence of norms or the causal relationship between norm perception and behaviour. Research on the effects of social norms relying on longitudinal or experimental methods allowing claims about the influence of norm manipulations are under-represented in the field [40]. Accordingly, we designed a mixed-design study to examine the effects of dynamic norms on meat consumption using different formats.

## 3. Main study

### 3.1. Method

#### 3.1.1. Sampling plan

We planned to collect data from a minimum of 100 participants in each between-subject condition (500 participants in total). We used a Bayesian stopping rule for data collection, and planned to stop collecting data when a threshold of *B* < 1/5 or *B* > 5 was reached for the study hypotheses [41], or when we reached a final sample of 1500 participants. We collected data at 100-participant intervals until we reached our specified thresholds. We detected multivariate outliers in the data using a robust Mahalanobis distance based on the minimum covariance determinant with a breakdown point of 0.25, and a chi-square at *p* = .001 ([38,39]; see also [42]). Participants who were identified as multivariate outliers on measured meat consumption outcomes (attitude, interest, intention and estimates of consumption) in the first wave were excluded from the sample at both time points.

#### 3.1.2. Participants

Participants were recruited from Prolific and redirected to the survey hosted on Qualtrics. After excluding participants who started the study more than once (*n* = 10, total of 22 cases), a total of 1508 cases remained. Of the 1508 unique participants who took part in the study, 1250 completed the second survey (82.89% completion rate). The pre-screening criteria used in the pilot study were also applied to the main study. The following were excluded from the study sample, in order (i) participants who started but did not complete the survey (*n* = 19); (ii) participants who were vegan/vegetarian (*n* = 30); (iii) participants who spent less than 5 s on the reading task (*n* = 57); and (iv) participants who were identified as multivariate outliers (*n* = 108). The final sample consisted of 1294 participants. Participants were mostly female (55.8%), ranging in ages 18–77 years (*M* = 39.97, s.d. = 13.71). Informed consent was obtained from all participants, and their data were identifiable via IDs generated by Prolific for the purpose of the study. Participants in the first wave were invited to the second and final waves of the study using Prolific’s internal messaging system. Participants were paid the equivalent of £6.00 h^−1^ at the first time point, then £7.50 h^−1^ at the second time point.

#### 3.1.3. Design and procedure

The study was presented as a survey on eating behaviour. The study used a 2 × 2 mixed design (norm type [dynamic/static] × visual cue [present/absent]), with a follow-up assessment after one week for the primary analyses, and included an additional control group with no normative information provided for non-crucial tests. Participants completed the measure of actual meat consumption, then they were randomly allocated to one of the five conditions. Participants then proceeded to the remaining outcome measures, followed by demographic questions. After one week, participants completed the outcome measures again. Participants were debriefed at the conclusion of the study.

To create norm statements, we relied on estimates provided by participants from the same sample population in a previous unpublished study (https://osf.io/gq6s3/). Specifically, we used the average estimate of the current percentage of British people reducing their meat consumption provided by participants in a control condition. This was estimated at 32.52%, which is close to other estimates used in dynamic norm research in the context of meat consumption [17,29], as well as national estimates of people limiting their meat consumption in recent years ([43]; e.g. [21,44]). Accordingly, we used 33% as the current estimate of people limiting their meat consumption.

#### 3.1.4. Materials

##### 3.1.4.1. Normative information

*Text*. Participants in the dynamic norm condition read the following text:

More and more people in the UK are changing. In 2020, 33% of British people—a figure increasing every year over the previous 5 years—successfully engaged in one or more of the following behaviours to eat less meat:

— Eating small portions of meat
— Opting out of eating meat several days of the week
— Adopting a vegan/vegetarian diet
— Taking part in Veganuary-style events.

Participants in the static norm condition read the following text:

In 2020, 33% of British people—roughly the same figure as in the previous 5 years—successfully engaged in one or more of the following behaviours to eat less meat:

— Eating small portions of meat
— Opting out of eating meat several days of the week
— Adopting a vegan/vegetarian diet
— Taking part in Veganuary-style events.

**Figure 1.**
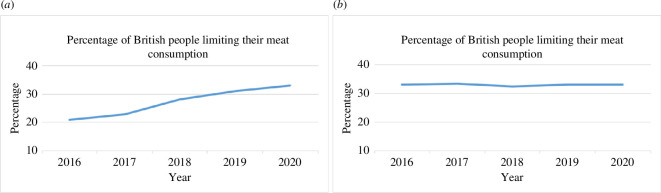
Visual cues used in visual dynamic condition (*a*) and visual static condition (*b*).

*Visual cues*. In conditions with an additional visual cue, participants saw a line graph showing the percentage of British people limiting their meat consumption from 2016 to 2020. In the static norm condition, the graph depicted a stable trend averaging about 33% every year. In the dynamic norm condition, the graph depicted an increasing trend of people decreasing their meat consumption, starting at roughly 20% in 2016, reaching about 33% in 2020 (see figure 1).

##### 3.1.4.2. Meat consumption outcomes

*Attitude*. Participants responded to the statement ‘My attitude towards eating less meat is…’ adapted from Fishbein and Ajzen [45] on a slider scale of 0 (*extremely unfavourable*) to 100 (*extremely favourable*).

*Interest*. Participants answered a single-item measure of interest in reducing meat consumption adapted from Sparkman and Walton [17], ‘How interested are you in eating less meat?’ on a slider scale of 0 (*not at all interested*) to 100 (*extremely interested*).

*Intention*. Participants responded to the statement ‘I intend to eat less meat within the next week’ adapted from Fishbein and Ajzen [45] on a slider scale of 0 (*strongly disagree*) to 100 (*strongly agree*). Participants also answered an additional single-item exploratory measure of intentions to reduce meat consumption adapted from Sparkman *et al.* [33]: ‘Do you intend to increase or to decrease your meat consumption over the next week (7 days)’ on a slider scale of 0 (*greatly decrease*) to 100 (*greatly increase*).

*Actual meat consumption*. Participants completed the food frequency questionnaire (FFQ) adapted from Sparkman *et al.* [33], covering consumption of all food groups over the period of one week. The scale has been adapted to a one week measurement period (*Never, Once (during those 7 days), twice, three times, four times, five times, six times, seven times, two times per day, three times per day, four or more times per day*). The sum of servings across meat groups was used to measure this outcome. Outliers on the FFQ were detected using the median absolute deviation (MAD). Responses above 3 MAD on the FFQ index of meat servings were excluded from analyses on the FFQ.

*Estimates of consumption*. Participants were asked to estimate the percentage of British people they think are eating less meat this year, next year and 6 years from now on a 0–100% slider scale (*α* = .91). The exact value participants chose was displayed onscreen above the slider.

##### 3.1.4.3. Checks

*Careless responses*. To detect low-quality responding, we checked response time in seconds per item (SPI; [46]). SPI is correlated with other validity indicators such as statistically improbable responses, disqualification in pre-screening and unusual responses to open-ended questions [47]. Based on an average reading speed of about 240–260 words min^−1^, the text prompts should take roughly 13 s to read [48]. Accordingly, we assumed that 5 s is a conservative estimate of minimum reading time, and we excluded the data of participants who spent less than 5 s on the reading task.

*Manipulation check*. Participants were asked ‘What has happened to meat consumption in the last 5 years?’ (*more people are eating less meat, people are eating about the same amount of meat, fewer people are eating less meat*). The manipulation was considered successful in the following conditions: (i) participants in the dynamic norm conditions selected ‘more people are eating less meat’ and (ii) participants in the static norm conditions selected ‘people are eating about the same amount of meat’. We reported analyses conducted without those who failed the manipulation check separately in the electronic supplementary material, table S1.

##### 3.1.4.4. Demographic questions

Participants were asked to report their political position on a 1–7 scale (1 = *very left wing*, 7 = *very right wing*). They also reported their age, gender and if they are vegan/vegetarian.

### 3.2. Data analyses

Details of preregistered hypotheses are presented in Appendix A.

#### 3.2.1. Analysis plan

Hypothesis 1: Making information about dynamic norms in relation to reduced meat consumption in the UK salient will lead to more positive effects on meat consumption outcomes than does making static norm information salient.

We tested this hypothesis using direct contrasts comparing the combination of dynamic norm experimental conditions against the combination of static norm control conditions for each measured meat consumption outcome at time 1.


(μvisual.dynamic,μtext.dynamic)>(μvisual.static,μtext.static)

Hypothesis 2: Including a visual cue will accentuate the difference between the dynamic norm and static norm conditions in meat consumption outcomes.

We tested this hypothesized interaction by comparing the differences between conditions using visual cue, and differences between conditions using text only for each measured outcome at time 1 using an interaction term [49].


(μvisual.dynamic−μvisual.static)>(μtext.dynamic−μtext.static)

Hypothesis 3: Including a visual cue will lead to a greater effect of dynamic norm information on meat consumption outcomes.

We tested this hypothesized simple main effect by comparing the effect of the dynamic norm with the visual cue against the dynamic norm without the visual cue for each measured outcome at time 1.


(μvisual.dynamic)>(μtext.dynamic)

Hypothesis 4: Dynamic norm information will positively influence meat consumption outcomes over a period of one week.

We tested this hypothesis by comparing between conditions difference on each pre- to post-test difference in score.


(μdynamic.post−μdynamic.pre)>(μstatic.post−μstatic.pre)

Hypothesis 5: Including a visual cue will accentuate the difference between the dynamic norm and static norm conditions in meat consumption outcomes over a period of one week.

We tested this interaction by comparing the differences between conditions using a visual cue, and the differences between conditions using text only for each measured outcome from time 1 to time 2.


(μvisual.dynamic.diff−μvisual.static.diff)>(μtext.dynamic.diff−μtext.static.diff)

Hypothesis 6: Including a visual cue will increase the effect of dynamic norm information on meat consumption outcomes over a period of one week.

We tested this hypothesized simple main effect by comparing the effect of the dynamic norm with the visual cue against the dynamic norm without the visual cue for each measured outcome from time 1 to time 2.


(μvisual.dynamic.diff)>(μtext.dynamic.diff)

We conducted all analyses using the statistical software R. We conducted direct contrasts to test all hypotheses rather than omnibus tests. We used Bayes factors to make any inferences about the hypotheses. Bayes factors are advantageous for several reasons: (i) they allow us to place probabilities on models that are updated using data; (ii) they quantify support for the null and the alternative hypothesis; (iii) they allow us to distinguish between null effects and insufficient data; and (iv) they make optional stopping possible without inflating type 1 error rates [41]. Although Bayes factors are continuous measures of evidence, we made inferences about our hypotheses using a threshold of *B* > 3 or *B* < 1/3, reflecting moderate strength of evidence [50].

#### 3.2.2. Missing data

We designed the surveys to force responses on all items, so we expected missing data to occur only in measured outcomes at the second time measurement point due to participant attrition. We used multiple imputations to generate and analyse 100 multiply imputed datasets over 10 iterations to maximize power for small effect sizes [51,52]. Multiple imputation methods are considered superior to other methods, such as listwise or pairwise deletion, or mean imputation, which can bias results and decrease statistical power and accuracy [53,54]. Incomplete variables were imputed within subsets reflecting the interaction between norm type and visual cue [55], under fully conditional specification method [56], with predictive mean matching implemented in the ‘mice’ package in R [57]. As recommended, we imputed missing outcome values at the item level, using the entirety of available data [58–60]. Around 15–16% of values were missing across measured outcomes at T2. The parameters of interest were estimated in each imputed dataset, and the pooled estimates and s.e. obtained using Rubin’s rules were used for the calculation of all Bayes factors [61]. For comparison, we also performed a complete case analysis, and the results were reported in the electronic supplementary material, table S2.

#### 3.2.3. Models of H_1_

We used half-normal distributions for all models of H_1_ across hypotheses, where the mode is set to 0, and the s.d. is set to the expected effect. This assumes directional predictions and that smaller effects are more probable than larger effects [62]. Bayes factors were notated as *B*_HN(0,*x*)_ where HN indicates that the model is half-normal, *x* is a scale factor of the expected effect and 0 represents the mode of the distribution. We estimated the expected effect using the results of previous studies, in combination with the results of our pilot study. All reported Bayes factors represent evidence for H_1_ over H_0_. In any reported exploratory analyses, we calculate post hoc Bayes factors testing the relative evidence for a hypothesis pointing in the opposite direction, notated as H_2_.

Previous studies found mean differences between dynamic and static norm conditions ranging from 0.6 to 0.78 units on a 1–7 Likert scale measuring interest in reducing meat consumption. This is equivalent to 9.86% on a 0–100% scale. Conversely, Aldoh *et al.* [29] found (no) difference of 0.03 units on a 1–7 Likert scale, equivalent to 0.43%. In our pilot, we found a mean difference of 4.15% between conditions. Based on the range of differences found, we expect to find a difference between conditions of roughly 5%.

The average difference between dynamic and static norm conditions in measured outcomes was similar across outcomes measured in the pilot, and therefore we used the same prior for all outcomes in the main study, apart from self-reported meat consumption. Sparkman *et al.* [33] found a dynamic norm appeal to reduce meat consumption resulted in a 6.8% reduction one month from baseline in self-reported meat consumption relative to a control condition. This is roughly equivalent to a difference of one serving reduction between groups. Similarly, we expected to find a difference in reduced meat consumption of one serving between dynamic and static norm conditions.

#### 3.2.4. Sensitivity analyses

We also reported *robustness regions* for all Bayes factors, indicating the range of prior scale factors that would lead to the same conclusion. Robustness regions are notated as RR [min, max], where min indicates the smallest s.d. and max indicates the largest s.d. that would result in the same conclusion.

### 3.3. Results

#### 3.3.1. Overview

The randomization check showed no significant differences in any demographic variables across conditions (*p*s > .23), and there were no significant differences in baseline meat consumption (*p* = .143). Means, s.d. and correlations of all study measures are displayed in table 3.

**Table 3.** Means, standard deviations and correlations between study measures.

measure	*n*	*M*	s.d.	correlations								
				1	2	3	4	5	6	7	8	9
interest T1	1294	46.76	29.14	—								
attitude T1	1294	56.02	26.98	.81**	—							
intention T1 (unipolar)	1294	48.36	30.29	.91**	.81**	—						
intention T1 (bipolar)	1294	50.16	14.64	−.45**	−.38**	−.45**	—					
interest T2	1098	48.24	28.50	.82**	.74**	.81**	−.37**	—				
Attitude T2	1098	57.33	26.78	.65**	.66**	.66**	−.31**	.71**	—			
Intention T2 (unipolar)	1098	50.49	29.60	.80**	.75**	.83**	−.36**	.88**	.70**	—		
Intention T2 (bipolar)	1098	49.76	15.29	−.39**	−.32**	−.38**	.52**	−.39**	−.29**	−.41**	—	
Projected consumption	1294	41.22	16.00	.31**	.30**	.31**	−.08**	.30**	.23**	.32**	−.07*	—
FFQ meat change	1092	−1.30	4.30	−.03	−.03	−.04	.07*	−.05	−.05	−.05	.06*	.03

Note: *N* = 1294.

*p < .05; **p < .01.

#### 3.3.2. Confirmatory analyses

An overview of descriptive statistics of the outcomes by condition is presented in table 4, and the results of hypothesis tests at time 1 are presented in table 5.

**Table 4.** Means and standard errors of outcomes in all study conditions at T1.

condition	*n*	attitude		interest		intention	
		*M*	s.e.	*M*	s.e.	*M*	s.e.
visual dynamic	262	58.82	1.76	49.43	1.85	51.35	1.91
text dynamic	260	52.13	1.62	42.52	1.71	43.56	1.79
visual static	261	57.28	1.69	47.22	1.87	47.89	1.89
text static	259	56.82	1.63	47.81	1.75	51.44	1.86
none	252	55.19	1.68	46.76	1.87	47.75	1.93

**Table 5.** Results of regression models and Bayes factors for each preregistered hypothesis at T1.

outcome	contrast	*b*	s.e.	d.f.	95% CI	*t*	*p*	inference		
								*B*_HN(0,5%)_	RR	conclusion^a^
H1										
attitude	static versus dynamic	−1.56	1.67	1291	[−4.84, 1.72]	−0.93	.351	0.17	[2.45, 10]	H_0_
interest	static versus dynamic	−1.52	1.81	1291	[−5.07, 2.02]	−0.84	.399	0.2	[2.8, 10]	H_0_
intention	static versus dynamic	−2.19	1.88	1291	[−5.87, 1.50]	−1.16	.244	0.17	[2.45, 10]	H_0_
attitude ^b^	none versus dynamic	0.30	2.07	1291	[−3.76, 4.36]	0.14	.886	0.42	[0.05, 6.55]	No
interest^b^	none versus dynamic	−0.77	2.24	1291	[−5.16, 3.62]	−0.34	.731	0.32	[4.8, 10]	H_0_
intention^b^	none versus dynamic	−0.28	2.32	1291	[−4.84, 4.28]	−0.12	.905	0.39	[0.05, 5.9]	None
H2										
attitude	interaction	6.24	3.34	1289	[−0.31, 12.78]	1.87	.062	3.49	[3.25, 9.55]	H_1_
interest	interaction	7.50	3.61	1289	[0.42, 14.58]	2.08	.038	4.6	[2.6, 10]	H_1_
intention	interaction	11.35	3.74	1289	[4.01, 18.69]	3.03	.002	22.41	[1.5, 10]	H_1_
H3										
attitude	text versus visual dynamic	6.70	2.36	1289	[2.07, 11.32]	2.84	.005	22.81	[1.05, 10]	H_1_
interest	text versus visual dynamic	6.91	2.55	1289	[1.91, 11.91]	2.71	.007	16.54	[1.2, 10]	H_1_
intention	text versus visual dynamic	7.79	2.64	1289	[2.61, 12.98]	2.95	.003	27.5	[1.1, 10]	H_1_

Note: *N* = 1294.

^a^ H_0_ = evidence for null hypothesis; None = no conclusion; H_1_ = evidence for alternative hypothesis.

^b^ Non-crucial tests.

*b*, raw regression slope; B, Bayes factor; CI, confidence interval; RR, robustness region.

##### 3.3.2.1. Hypothesis 1


(μvisual.dynamic,μtext.dynamic)>(μvisual.static,μtext.static)

We hypothesized that making information about dynamic norms in relation to reduced meat consumption in the UK salient would lead to more positive effects on meat consumption outcomes than would making static norm information salient. Specifically, we expected that participants who viewed dynamic norm information would have more positive attitudes (H1a), higher interest (H1b) and higher intentions (H1c) towards reducing their meat consumption at time 1 compared with participants who viewed static norm information. We found moderate evidence for an absence of an effect of dynamic norms (versus static norms) on attitude (*b* = −1.56, s.e*.* = 1.67, *B*_HN(0,5%)_ = 0.17, RR [2.45, 10]), interest (*b* = −1.52, s.e*.* = 1.81, *B*_HN(0,5%)_ = 0.20, RR [2.8, 10]) and intention (*b* = −2.19, s.e*.* = 1.88, *B*_HN(0,5%)_ = 0.17, RR [2.45, 10]). Therefore, H1a–H1c were not supported.

##### 3.3.2.2. Hypothesis 2


(μvisual.dynamic−μvisual.static)>(μtext.dynamic−μtext.static)

We hypothesized that including a visual cue would accentuate the difference between the dynamic norm and static norm conditions in meat consumption outcomes. Specifically, we expected visual cues to accentuate the difference between dynamic and static norm conditions in participants’ attitudes (H2a), interest (H2b) and intentions (H2c) towards reducing their meat consumption. We found moderate evidence for an interaction effect between norm type and visual cue on attitude (*b* = 6.24, s.e*.* = 3.34, *B*_HN(0,5%)_ = 3.49, RR [3.25, 9.55]) and interest (*b* = 7.50, s.e*.* = 3.61, *B*_HN(0,5%)_ = 4.60, RR [2.6, 10]). We also found strong evidence for an interaction effect on intention (*b* = 11.35, s.e*.* = 3.74, *B*_HN(0,5%)_ = 22.41, RR [1.50, 10]).

To probe the interactions, we conducted unregistered post hoc simple-effects analysis examining the difference between (i) static norm/visual cue absent versus dynamic norm/visual cue absent and (ii) static norm/visual cue present versus dynamic norm/visual cue present. We computed two post hoc Bayes factors for each comparison reflecting a positive effect of dynamic norms over static norms within each simple-effects analysis (H_1_), or a positive effect of static norms over dynamic norms (H_2_). When visual cues were absent, we found evidence against a positive effect of dynamic norms versus static norms (H_1_) but evidence for a positive effect of static norms versus dynamic norms (H_2_) on attitudes (*b* = −4.69, s.e*.* = 2.36, H_1_: *B*_HN(0,5%)_ = 0.15, RR [2.1, 10], H_2_: *B*_HN(0,5%)_ = 4.11, RR [1.9, 9.45]), interest (*b* = −5.29, s.e*.* = 2.56, H_1_: *B*_HN(0,5%)_ = 0.16, RR [2.2, 10], H_2_: *B*_HN(0,5%)_ = 4.79, RR [1.9, 10]) and intentions (*b* = −7.88, s.e*.* = 2.65, H_1_: *B*_HN(0,5%)_ = 0.13, RR [1.7, 10], H_2_: *B*_HN(0,5%)_ = 29.15, RR [1.1, 9.45]).

When visual cues were present, evidence was insensitive for tests of a positive effect of dynamic norms versus static norms (H_1_) but we found evidence against a positive effect of static norms versus dynamic norms (H_2_) on attitude (*b* = 1.54, s.e*.* = 2.36, H_1_: *B*_HN(0,5%)_ = 0.73, RR [0.05, 10], H_2_: *B*_HN(0,5%)_ = 0.28, RR [4.1, 10]), interest (*b* = 2.21, s.e*.* = 2.55, H_1_: *B*_HN(0,5%)_ = 0.95, RR [0.05, 10], H_2_: *B*_HN(0,5%)_ = 0.27, RR [3.9, 10]) and intention (*b* = 3.47, s.e*.* = 2.64, H_1_: *B*_HN(0,5%)_ = 1.6, RR [0.05, 10], H_2_: *B*_HN(0,5%)_ = 0.22, RR [3.2, 10]). In other words, our second exploratory simple-effects analysis suggests that there was no evidence to suggest a (non) positive effect of dynamic norms (versus static norms) with visual cues, but there was moderate evidence suggesting an absence of a positive effect of static norms (versus dynamic norms) with visual cues on all outcomes.

**Figure 2.**
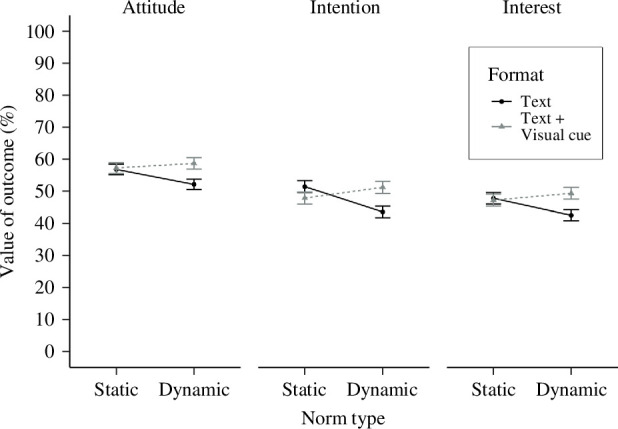
Means of outcomes by norm type and visual cue at T1. Note. Error bars represent standard errors.

Thus, we found the hypothesized interactions between norm type and visual cue, and the pattern of effects meant that hypotheses were supported. However, support for the hypotheses seemed to be predicated on the lower scores of the dynamic norm condition than the static norm condition when only textual information was provided (i.e. in the absence of a visual cue). These results suggest that there may be a negative effect of using text-only information for providing dynamic norm information, compared with static norm information. Means and standard errors of outcomes at T1 are shown in figure 2.

##### 3.3.2.3. Hypothesis 3


(μvisual.dynamic)>(μtext.dynamic)

We hypothesized that including a visual cue would lead to a greater effect of dynamic norm information on meat consumption outcomes. Specifically, we expected participants who view dynamic norm information accompanied by a visual cue will report more positive attitudes (H3a), higher interest (H3b) and higher intentions (H3c) towards reducing their meat consumption than will participants who view dynamic norm information without a visual cue. We found strong evidence for a simple effect of visual cue within dynamic norm conditions, whereby dynamic norms with a visual cue had a more positive effect than did dynamic norms without a visual cue, on attitude (*b* = 6.70, s.e*.* = 2.36, *B*_HN(0,5%)_ = 22.81, RR [1.05, 10]), interest (*b* = 6.91, s.e*.* = 2.55, *B*_HN(0,5%)_ = 16.54, RR [1.2, 10]) and intentions (*b* = 7.79, s.e*.* = 2.64, *B*_HN(0,5%)_ = 27.5, RR [1.1, 10]). Therefore, H3a–H3c were supported.

##### 3.3.2.4. Hypothesis 4


(μdynamic.T2−μdynamic.T1)>(μstatic.T2−μstatic.T1)

The means and standard errors of all measured outcomes at T2 are presented in table 6, and the results of hypothesis tests at time 2 are presented in . We hypothesized that dynamic norm information would positively influence meat consumption outcomes over a period of one week. Specifically, we expected a greater positive change in participants’ attitudes (H4a), interest (H4b), intentions (H4c) and reduced self-reported meat consumption (H4d). We found moderate evidence for an absence of an effect of dynamic norms (versus static norms) on attitude change (*b* = −2.22, s.e*.* = 1.47, *B*_HN(0,5%)_ = 0.12, RR [1.6, 10]), interest change (*b* = −0.42, s.e*.* = 1.17, *B*_HN(0,5%)_ = 0.17, RR [2.5, 10]) and change in self-reported meat consumption (*b* = 0.04, s.e*.* = 0.22, *B*_HN(0,1)_ = 0.18, RR [0.55, 5]). Evidence remained insensitive for intentions change (*b* = 1.33, s.e*.* = 1.17, *B*_HN(0,5%)_ = 0.73, RR [0.05, 10]). Therefore, H4a, H4b and H4d were not supported, and we suspend judgement on H4c.

**Table 6.** Means and standard errors of outcomes in all study conditions at T2.

condition	*n*	attitude		interest		intention		meat servings^a^	
		*M*	s.e.	M	s.e.	*M*	s.e.	*M*	s.e.
visual dynamic	262	56.05	1.74	49.55	1.86	52.54	1.90	7.02	0.21
text dynamic	260	54.27	1.65	45.26	1.77	47.94	1.82	6.86	0.23
visual static	261	57.61	1.74	47.38	1.78	50.53	1.86	6.80	0.20
text static	259	59.63	1.54	51.03	1.66	52.42	1.76	6.68	0.23
none	252	57.44	1.67	48.35	1.85	50.09	1.87	7.00	0.25

Note: Descriptive statistics are generated from a complete imputed dataset randomly selected from 100 imputed datasets generated.

^a^ Excluding participants who were outliers on the FFQ. *n*s = 245, 243, 251, 248, 238, respectively.

**Table 7.** Pooled results of regression models and Bayes factors for each preregistered hypothesis at T2.

outcome	contrast	*b*	s.e.	d.f.	95% CI	*t*	*p*	inference		
								*B*_HN_	RR	conclusion^a^
H4										
attitude change	static versus dynamic	−2.22	1.47	1291	[−5.11, 0.66]	−1.51	.131	0.12	[1.6, 10]	H_0_
interest change	static versus dynamic	−0.42	1.17	1291	[−2.73, 1.88]	−0.36	.720	0.17	[2.5, 10]	H_0_
intention change	static versus dynamic	1.33	1.17	1291	[−0.96, 3.63]	1.14	.254	0.73	[0.05, 10]	None
FFQ meat change	static versus dynamic	0.04	0.22	1222	[−0.38, 0.47]	0.21	.836	0.18	[0.55, 5]	H_0_
attitude change	none versus dynamic	−3.59	1.85	1291	[−7.22, 0.03]	−1.95	.052	0.12	[1.7, 10]	H_0_
interest change	none versus dynamic	−0.98	1.44	1291	[−3.81, 1.85]	−0.68	.498	0.17	[2.5, 10]	H_0_
intention change	none versus dynamic	0.28	1.43	1291	[−2.54, 3.09]	0.19	.847	0.32	[4.8, 10]	H_0_
FFQ meat change	none versus dynamic	−0.21	0.27	1222	[−0.75, 0.33]	−0.75	.452	0.53	[0.05, 1.6]	None
H5										
attitude change	interaction	−3.11	2.92	1289	[−8.85, 2.62]	−1.06	.287	0.27	[4, 10]	H_0_
interest change	interaction	0.35	2.34	1289	[−4.25, 4.94]	0.15	.882	0.47	[0.05, 7.45]	None
intention change	interaction	−4.56	2.31	1289	[−9.10, −0.02]	−1.97	.049	0.15	[2.1, 10]	H_0_
FFQ meat change	interaction	0.06	0.43	1220	[−0.79, 0.91]	0.15	.884	0.36	[0.05, 1.05]	None
H6										
attitude change	text versus visual dynamic	−5.23	2.05	1289	[−9.24, −1.21]	−2.55	.011	0.11	[1.5, 10]	H_0_
interest change	text versus visual dynamic	−2.29	1.61	1289	[−5.45, 0.88]	−1.42	.157	0.13	[1.85, 10]	H_0_
intention change	text versus visual dynamic	−3.37	1.61	1289	[−6.54, −0.21]	−2.09	.037	0.1	[1.4, 10]	H_0_
FFQ meat change	text versus visual dynamic	−0.06	0.30	1220	[−0.66, 0.53]	−0.20	.840	0.34	[0.05, 1]	None

Note: *N* = 1294

^a^ H_0_ = evidence for null hypothesis, None = no conclusion, H_1_ = evidence for alternative hypothesis.

*b*, raw regression slope; B, bayes factor; CI, confidence interval; RR, robustness region.

##### 3.3.2.5. Hypothesis 5


(μvisual.dynamic.diff−μvisual.static.diff)>(μtext.dynamic.diff−μtext.static.diff)

We hypothesized that including a visual cue would accentuate the difference between the dynamic norm and static norm conditions in meat consumption outcomes over a period of one week. Specifically, we expected that visual cues would accentuate the difference in attitudes’ change (H5a), interest change (H5b), intentions’ change (H5c) and self-reported meat consumption (H5d) between time 1 and time 2. We found moderate evidence for an absence of an interaction effect between norm type and visual cue on attitude change (*b* = −3.11, s.e*.* = 2.92, *B*_HN(0,5%)_ = 0.27, RR [4, 10]) and intentions change (*b* = −4.56, s.e*.* = 2.31, *B*_HN(0,5%)_ = 0.15, RR [2.1, 10]). Evidence remained insensitive for interest change (*b* = 0.35, s.e*.* = 2.34, *B*_HN(0,5%)_ = 0.47, RR [0.05, 7.45]), and change in self-reported meat consumption (*b* = 0.06, s.e*.* = 0.43, *B*_HN(0, 1)_ = 0.36, RR [0.05, 1.05]). Therefore, H5a and H5c were not supported, and we suspend judgement on H5b and H5d.

##### 3.3.2.6. Hypothesis 6


(μvisual.dynamic.diff)>(μtext.dynamic.diff)

We hypothesized that including a visual cue would increase the effect of dynamic norm information on meat consumption outcomes over a period of one week. Specifically, we expected that participants who viewed dynamic norm information accompanied by a visual cue would show more positive attitude change (H6a), interest change (H6b), intentions change (H6c) and reduced self-reported meat consumption (H6d) compared with participants who viewed dynamic norm information without the visual cue (between time 1 and time 2). We found moderate evidence for an absence of a positive effect of a visual cue within dynamic norm conditions on attitude change (*b* = −5.23, s.e. = 2.05, *B*_HN(0, 5%)_ = 0.11, RR[1.5, 10]), interest change (*b* = −2.29, s.e. = 1.61, *B*_HN(0, 5%)_ = 0.13, RR[1.85, 10]), intentions change (*b* = −3.37, s.e. = 1.61, *B*_HN(0, 5%)_ = 0.1, RR[1.4, 10]). Evidence remained insensitive for change in self-reported meat consumption (*b* = −.06, s.e. = .30, *B*_HN(0, 1)_ = 0.34, RR[0.05, 1]). Therefore, hypotheses H6a–H6c were not supported, and we suspend judgement on hypothesis H6d.

### 3.3.3. Unregistered secondary analyses

We conducted additional analyses to examine between-group differences in measured outcomes (rather than changes in outcomes) at T2 (see electronic supplementary material, table S3). However, there was no positive effect of dynamic norms (versus static norms) on any measured outcomes (*B*s < 0.27). There was an interaction of norm type and visual cue on interest and intentions to reduce meat consumption (*B*s > 3.11).

To probe the interaction effects on interest and intentions, we conducted unregistered post hoc simple-effects analysis similar to those reported under H2, using two one-directional opposing models (H_1_ and H_2_). We examined the difference between (i) static norm/visual cue absent versus dynamic norm/visual cue absent; and (ii) static norm/visual cue present versus dynamic norm/visual cue present (see electronic supplementary material, table S4). When visual cues were absent, we found evidence against a positive effect of dynamic norms versus static norms (H_1_), but evidence for a positive effect of static norms versus dynamic norms (H_2_) on interest (H_1_: *B* = 0.15, H_2_: *B* = 7.23). Evidence remained insensitive for effects on intention (H_1_: *B* = 0.20, H_2_: *B* = 2.36). These results suggest a potentially detrimental effect of using text-only information to communicate dynamic norms, on interest in reducing meat consumption.

When visual cues were present, evidence was insensitive for tests of a positive effect of dynamic norms versus static norms (1/3 < H_1_ *B*s < 3), but we found evidence against a positive effect of static norms versus dynamic norms (H_2_) on interest and intention (H_2_ *B*s < 3). Altogether, our second exploratory simple-effects analysis suggests that there was no evidence to suggest a (non) positive effect of dynamic (versus static) norms with visual cues on interest and intention, but there was moderate evidence suggesting an absence of a positive effect of static norms (versus dynamic norms) with visual cues on interest and intention.

Participants who viewed dynamic norm information with a visual cue had higher interest in reducing their meat consumption than those who viewed dynamic norm information in the form of text only (*B* = 3.14). All other results did not reach sufficient evidential thresholds (1/3 < *B* < 3).

## 4. Discussion

We hypothesized that dynamic norms would have a positive effect on meat consumption outcomes, in comparison with static norms (H1a–H1c); however, we found evidence for an absence of a main effect of dynamic (versus static) norms on attitude towards reducing meat consumption, interest in reducing meat consumption and intentions to reduce meat consumption. We hypothesized that visual cues would accentuate the differences between static and dynamic norm information (H2a–H2c); we found moderate to strong evidence for an interaction between norm type and visual cue on attitudes, interest and intentions to reduce meat consumption, where the difference between dynamic norms conditions with and without a visual cue (i.e. (μvisual.dynamic−μvisual.static) , was larger than the difference between static norm conditions with and without a visual cue (i.e. (μtext.dynamic−μtext.static) ). Our post hoc exploratory simple-effects analyses suggest a detrimental effect of using text only when providing dynamic norm information compared with when providing static norm information. We hypothesized that including a visual cue would improve the effect of dynamic norm information on meat consumption outcomes (H3a–H3c). We found strong evidence that participants who viewed dynamic norm information alongside a visual cue had more positive attitudes, interest and intentions to reduce meat consumption than did participants who viewed dynamic norm information without a visual cue. We hypothesized that dynamic norms (versus static norms) would have a positive effect on meat consumption outcomes over a period of one week (H4a–H4d). There was moderate evidence for the absence of a positive effect of dynamic norms on changes in attitude, interest and self-reported meat consumption over a period of one week (H4a, H4b and H4d). There was no evidence for or against an effect on changes in intention (H4c). We hypothesized that including a visual cue would accentuate the difference between the dynamic norm and static norm conditions in meat consumption outcomes over a period of one week (H5a–H5d). There was moderate evidence for an absence of an interaction effect between norm type and visual cue on changes in attitude, and intentions over a period of one week. There was no evidence for or against an interaction effect on changes in interest and self-reported meat consumption over a period of one week (H5b and H5d). Finally, we hypothesized that including a visual cue would increase the effect of dynamic norm information on meat consumption outcomes over a period of one week. We found moderate evidence for an absence of a positive effect of a visual cue within dynamic norm conditions on changes in attitude, interest and intentions (H6a–H6c). There was no evidence for or against a positive effect of visual cue within dynamic norm conditions on changes in self-reported meat consumption (H6d). Overall, the results showed a positive effect of including visual cues when conveying dynamic norm information, but evidence suggests that changes in participants who viewed visual dynamic norm information are not larger than those who did not over a short period of 7 days.

Dynamic norms had a larger effect on meat consumption outcomes when accompanied by a visual cue than when not. Some research has shown that people struggle with interpreting relative change and percentages more generally [63,64]. Visual cues may have increased effects by clearly signalling the change in prevalence over time, thereby stimulating rapid information processing. According to the dual processing theory, imagery and symbolism engage the experiential processing system, which encodes the world quickly and intuitively [65]. Our finding warrants further research to understand the exact mechanism explaining this effect. A future study may specifically examine participants’ interpretation of the norm with and without the visual cue.

Behaviour changes occur over time, and often unfold through a series of different stages, ranging from unwillingness to perform the behaviour, to eventually partaking in the desired behaviour [66,67]. The transtheoretical model of behaviour change assumes that change occurs over a series of stages, broadly moving from pre-contemplation (no intention to take actions, perhaps due to lack of motivation or information), to contemplation (considering costs and benefits of action), then preparation (planning to take action) and eventually enacting the behaviour [68,69]. This pattern of change towards desired behaviour has been demonstrated across a range of different behaviours, such as quitting smoking, using condoms, increasing exercise, healthy eating, among several others [70]. People’s perceptions of the costs and benefits associated with a certain behaviour can influence their progression through stages of behaviour change. Different motives and food-related attitudes can influence willingness to eat sustainably, such as by reducing meat consumption. Tobler *et al.* [67] examined the association between participants’ reported current stage (e.g. pre-contemplation, contemplation, etc.) and different motives for willingness to reduce meat consumption such as concerns for price, taste, health, environment and animals’ suffering. They found that health was the strongest predictor of participants being in the action stage, and lower likelihood of participants being in the pre-contemplation stage. Relatedly, Aldoh *et al*.’s [29] exploratory word cloud of participants’ responses when asked why they think others might be reducing their meat consumption showed that ‘health’ was the most frequently mentioned word (see electronic supplementary material, figure S2.1). Highlighting the health benefits of reducing meat alongside dynamic norm information may be a potential method for increasing positive effects on intentions, and subsequently likelihood of reaching the final stage of behaviour change.

Our results suggest that there was no difference between dynamic and static norm conditions in changes in self-reported meat consumption over a period of one week. Changing dietary behaviour to consume sustainably often requires individuals to overcome barriers, such as changing habits and lifestyle [67]. As a result, progress towards action can happen gradually, perhaps over a period longer than 7 days. Indeed, Carfora *et al.* [71] found that the effects of their dynamic norm intervention were stronger in the long term than the short term. Specifically, participants had reduced their meat consumption at T3, more than they had at T2 and T1. The authors speculate that this may be due to the additional time needed to reflect on provided information, and to translate this into consistent actions. Similarly, Sparkman *et al*.’s [4] results show dynamic norms appeals to reduce meat consumption led to a larger reduction of participants’ meat consumption in a fourth wave (8.8% relative to changes in the control condition) than in a third wave (6.8%). Taken together, these results suggest that the intervention may have potentially improved recursive processes that unfolded over time, or actual dietary change may take longer time to unfold. It is possible that differences between conditions may have changed further over time, but it is not possible to verify this without further research. Our secondary analyses suggest a generally positive interaction effect on interest and intentions, and a benefit of visual cues in dynamic norm information on interest at T2. Perhaps visual dynamic norm information had a general positive influence on some outcomes at the second measurement time point, but they did not drive changes above and beyond changes observed in dynamic norm information using text alone.

Initial changes in people’s dietary behaviour by reducing meat consumption may also pave the way for further reduction, and even elimination of meat consumption entirely in the future. Even short-term adherence (7 days) to a diet involving decreased meat consumption can lead to subsequent overall reductions in meat consumption [72]. Longer engagement with events such as ‘Meat Free Monday’ has been linked to an increased likelihood of becoming vegan/vegetarian, where roughly 20% of those engaged eliminated meat consumption entirely within 3–5 years [73]. Initial changes towards desired behaviours may lead to experienced benefits and increases in efficacy for performing the behaviour, thereby consolidating behaviour change [74,75]. A challenge lies in maintaining people’s active engagement in such events; exploring how different initiatives may attract and retain participants could be a fruitful area of research.

### 4.1. Limitations

This study tested the effect of a one-shot dynamic norm intervention. This is also the case in most published studies testing the effects of dynamic norms on the uptake of desired attitudes or behaviours. The study may have benefited from repeated provision of dynamic norm messages, varying in their representation of the changing norm. There could be an advantage of repeated exposure to dynamic norm messages over a single message. Carfora *et al.* [71] used a design where dynamic norm information was provided every day over a period of one month via a chatbot, but there is as yet no research directly comparing conditions with single versus repeated exposure to dynamic norm messages.

The materials used to convey dynamic norm information in text did not include explicit reference to the norm in the past, but the visual cue in the dynamic norm condition showed that the reduced meat consumption norm was about 20% in 2016. Future research could explore the effect of explicit textual reference in dynamic norm conditions to the past norm.

## 5. Conclusion

This study found that dynamic norms may be more potent if a visual cue is included, but there was no clear benefit here of using dynamic norm information with or without a visual cue over a short-term period of one week. Future research in this area can explore how elements of dynamic norm messaging can be improved to instigate further change over time.

## Data Availability

Data and relevant code for this research work are stored in GitHub [76] and have been archived within the Zenodo repository [77]. Supplementary material is available online [78].

## Appendix A

See table 8.

**Table 8.** Hypothesis registration table.

question	hypothesis	sampling plan	analysis plan	rationale for deciding the sensitivity of the test	interpretation given different outcomes	theory that could be shown wrong by the outcomes	outcome
do salient dynamic norms regarding reduced meat consumption in the UK lead to more positive effects on meat consumption outcomes than static norms?	H1: making information about dynamic norms in relation to reduced meat consumption in the UK salient will lead to more positive effects on meat consumption outcomes than does making static norm information salient.	we will use a Bayesian stopping rule where we will stop data collection when a threshold of *B* < 1/5 or *B* > 5 is reached for the study hypotheses comparing dynamic norm conditions to static norm conditions. We will recruit a maximum of 1500 participants due to funding restraints.	we will use contrast coding to compare dynamic norm conditions against static norm conditions. We will run analyses for each outcome: (i) attitudes, (ii) interest, and (iii) intention. We will use the raw difference in scores and the s.e. of the difference to calculate a Bayes factor for each outcome. We will model H_1_ using a half-normal distribution with a mode of 0 and s.d. of 5%. Non-crucial test: we will compare dynamic norm conditions to control condition using the same method.	a difference of 5% is a rough average of the differences found across outcomes in prior research. We use a Bayes factor threshold of *B* < 1/3 or *B* > 3, which is considered moderate evidence for the null or alternative hypotheses.	H_1_ is supported if a Bayes factor equal to or more than 3 is reached. H_0_ is supported if a Bayes factor less than or equal to 1/3 is reached. Bayes factors in between will not be considered evidence for either, and we will conclude that the results are insensitive.	dynamic norm information does not positively influence meat consumption outcomes, namely, attitudes towards meat consumption, interest in reducing meat consumption and intentions to reduce meat consumption. We can deduce that dynamic norm information is not effective in this context.	not supported
do visual cues accentuate the difference between dynamic norm and static norm information on meat consumption outcomes?	H2: including a visual cue will accentuate the difference between the dynamic norm and static norm conditions in meat consumption outcomes.	see above.	we will compare the differences between the conditions including a visual cue, to the differences between the conditions using text only using an interaction term. The corresponding raw interaction and its s.e. will be used to calculate a Bayes factor for each outcome: (i) attitudes, (ii) interest, and (iii) intention. We will model H_1_ using a half-normal distribution with a mode of 0 and s.d. of 5%.	we expect the difference between dynamic norm and static norm conditions using visual cues to be larger than the difference between dynamic and static norm conditions using text alone.	see above.	including a visual cue does not accentuate the difference between dynamic and static norm conditions in meat consumption outcomes.	supported
do visual cues influence the effect of dynamic norm information on meat consumption outcomes?	H3: including a visual cue will lead to a greater effect of dynamic norm information on meat consumption outcomes.	see above.	we will test the simple effect of format within the condition by contrasting the dynamic norm with visual cue condition to the dynamic norm with text-only condition. We will run analyses for each outcome: (i) attitude, (ii) interest, and (iii) intention. We will use the raw difference in scores and the s.e. of the difference to calculate a Bayes factor for each outcome. We will model H_1_ using a half-normal distribution with a mode of 0 and s.d. of 5%.	we expect a visual cue will increase the efficacy of dynamic norm information over and above text alone by about 5%. We use the same Bayes factor threshold specified above.	see above.	including a visual cue while depicting dynamic norm does not improve the efficacy of dynamic norm messaging.	supported
do salient dynamic norms regarding reduced meat consumption in the UK lead to more positive effects on meat consumption outcomes than static norms over a period of one week?	H4: dynamic norm information will positively influence meat consumption outcomes over a period of one week. H4a–H4c	see above.	at time 2, we will calculate the difference between pre-test and post-test scores on: (i) attitudes, (ii) interest, and (iii) intentions. We will then use contrast coding to compare the change in outcomes between dynamic norm conditions and static norm conditions. Non-crucial test: we will compare dynamic norm conditions to control condition using the same method.	a difference of 5% across outcomes. We use the same Bayes factor threshold specified above.	see above.	dynamic norms are not effective means of influencing cognitive meat consumption outcomes over a period of one week.	not supported: H4a, H4b Suspend judgement: H4c
	H4d	see above.	at time 2, we will calculate two new variables totalling all reported meat consumption at baseline and follow-up. We will then subtract the post-intervention meat consumption from baseline meat consumption. Finally, we will use contrast coding to compare the change in consumption between dynamic norm conditions and static norm conditions. We will model H_1_ with a mode of 0 and s.d. of 1 serving. Non-crucial test: we will compare dynamic norm conditions to control condition using the same method.	we expect that participants in the dynamic norm condition will have reduced their overall meat consumption by one serving more than participants in the static norm control. We use the same Bayes factor threshold specified above.	see above.	dynamic norms are not effective means of changing actual meat consumption over a period of one week.	not supported
do visual cues accentuate the difference between dynamic norm and static norm information on meat consumption outcomes over a period of one week?	H5: including a visual cue will accentuate the difference between the dynamic norm and static norm conditions in meat consumption outcomes over a period of one week.	see above.	We will compare the differences in changes between the conditions including a visual cue, to the differences in changes between the conditions using text only using an interaction term. The corresponding raw interaction and its s.e. will be used to calculate a Bayes factor for each outcome: (i) attitudes, (ii) interest, (iii) intention, and (iv) change in meat consumption. We will model H_1_ for cognitive meat consumption outcomes using a half-normal distribution with a mode of 0 and s.d. of 5%. For self-reported meat consumption change, we will model H_1_ with a mode of 0 and s.d. of 1 serving.	we expect the difference in changes between dynamic norm and static norm conditions using visual cues to be larger than the difference in changes between dynamic and static norm conditions using text alone.	see above.	including a visual cue does not accentuate the difference between dynamic and static norm conditions in meat consumption outcomes over a period of one week.	not supported: H5a, H5c Suspend judgement: H5b, H5d
Do visual cues influence the effect of dynamic norm information on meat consumption outcomes over a period of one week?	H6: including a visual cue will increase the effect of dynamic norm information on meat consumption outcomes over a period of one week.	see above.	we will test the simple effect of format within the condition by contrasting the dynamic norm with visual cue condition to the dynamic norm with text-only condition. We will run analyses for each outcome: (i) change in attitude; (ii) change in interest; (iii) change in intention; and (iv) change in meat consumption. We will use the raw difference in scores and the s.e. of the difference to calculate a Bayes factor for each outcome. We will model H_1_ for cognitive meat consumption outcomes using a half-normal distribution with a mode of 0 and s.d. of 5%. For self-reported meat consumption change, we will model H1 with a mode of 0 and s.d. of 1 serving.	a difference of 5% across changes in cognitive outcomes (attitudes, interest and intentions). Additionally, a difference of one serving for reported meat consumption. We use the same Bayes factor threshold specified above.	see above.	including a visual cue while depicting dynamic norm does not improve the efficacy of dynamic norm messaging over a period of one week.	not supported: H6a–H6c Suspend judgement: H5d
